# Ureteral injuries from gunshots and shells of explosive devices

**DOI:** 10.4103/0974-7796.62920

**Published:** 2010

**Authors:** Ammar Fadil Abid, Hussein Lafta Hashem

**Affiliations:** Al-Yarmouk Teaching Hospital, College of Medicine, The University of Mustanisriya, Baghdad, Iraq

**Keywords:** Gunshot, penetrating injury, shells of explosive devices, ureter

## Abstract

**Context:**

Penetrating rather than blunt trauma is the most common cause of ureteral injuries. The approach to management differs from the far more common iatrogenic injury.

**Aims:**

The purpose of this series is to report our experience in ureteral trauma management, with attention to the diagnosis, repair, and outcome of these injuries.

**Materials and Methods:**

From April 2003 to October 2009, all abdominal trauma cases received alive, reviewed for penetrating ureteric injuries

**Results:**

A total of twenty (fifteen male, five female) penetrating ureteral injuries were evaluated. All penetrating ureteric injuries were due to (9 gunshot and 11 shells from explosive devices). Since the patients had a clear indication for surgery, no IVU or CT scan was done preoperatively, major intra-abdominal injuries were often associated. The diagnosis of ureteric injury was made intraoperatively in 8 cases (40%) While, twelve cases (60%) were diagnosed postoperatively. Eight ureteric injuries (40%) were proximal 1/3, 4 (20%) to middle 1/3 and 8 (40%) to the distal 1/3. Management was with stenting in 2 patients, ureteroureterostomy in 8, ureteroneocystostomy in 6, and nephrectomy in 4.

**Conclusions:**

In this study, a delay in diagnosis was a contributory factor in morbidity related to ureteral injury, the need for second operation in already compromised patients from associated injuries, The presence of shock on admission, delayed diagnosis, and colon injuries were associated with a high complication rate. Ureteral injuries must be considered early during the evaluation of penetrating abdominal injuries.

## INTRODUCTION

Traumatic ureteral injuries are uncommon. Penetrating rather than blunt trauma is the most common cause of ureteral injuries.[1,2] The approach to management differs from the far more common iatrogenic injury.

The ureter is the least often injured genito-urinary organ accounting for less than 1% of all urologic traumas secondary to violence.[1‐3] Recently, the increased incidence of injuries of the ureter can be explained by an increase in armed violence, especially due to the explosive devices that inflict a large number of causalities. Visceral injuries are commonly associated with ureteral injury and are easily identified in most cases. These patients often have a high degree of mortality that approaches one third.[2] Patients with hemodynamic instability and extensive blood loss are more susceptible for ureteral lesions not identified at surgical exploration.[3‐7]

Ureteral injury is usually silent, producing no early signs or symptoms. Hematuria is typically absent on presentation, as described in several series, and urinalysis is normal in 15–55% of patients with ureteral injury.[1‐7]

The purpose of this series is to report our experience in ureteral trauma management, with attention to the diagnosis, repair, and outcome of these injuries.

## MATERIALS AND METHODS

From April 2003 to October 2009, all abdominal trauma cases admitted alive in the Emergency Department at Al-Yarmouk Hospital that is one of the primary sites in Baghdad for acute management of civilian trauma, were reviewed for penetrating ureteric injury.

Injuries were staged using the American Association for the Surgery of Trauma (AAST) organ injury severity scale.[2,8] The mechanism of injury as well as the number and severity of associated injuries were noted. Urgent surgical exploration was performed in most cases with gunshot wounds and blast injury penetrating the peritoneal cavity or retro peritoneum, without any further investigation including computed tomography (CT) scan or intravenous urography (IVU). Since we were dealing with multiorgan injured trauma victim patients with life-threatening concomitant injuries.

When ureteric injury was encountered or looked for during laparotomy (expected according to the injury type and abdominal wound site), the principle was to repair the ureter according to the site of injury. In the cases where the ureteric injury was missed and discovered early either postoperatively by excess fluid drainage, or lately as abdominal mass (urinoma), we did imaging studies and proceeded for trial of cystocopy and ureteric catheterization. In case ureteric stent was passed, we kept it for 4-6 weeks, otherwise we planned for exploration and repair. In cases present with a large urinom, we drained it by percutaneous abdominal drain followed by the same lines of treatment mentioned above.

All repairs included adequate debridement of ureteral margins, spatulation, interrupted suturing using a four or five zero absorbable suture, drain, bladder catheter drainage and antibiotic prophylaxis.

## RESULTS

A total of 20 (15 males and 5 females) cases of ureteral injuries were evaluated retrospectively. Age ranged from 16 to 60 years. The right ureter was involved in 13 (65%) patients and the left one in seven (35%) patients, and there were no bilateral lesions.

All penetrating ureteric injuries were due to bullets: 9 cases (45%) from pistols, rifles, and/or machine guns and 11 cases (55%) due to shells from explosive devices. None of the wounds resulted from knives.

Since the patients showed a clear indication for surgery, no IVU or computerized tomography (CT) scan was done preoperatively. So the diagnosis was according to the operative and postoperative findings like development of fistulae, hydronephrosis or urinoma.

**Figure 1:**
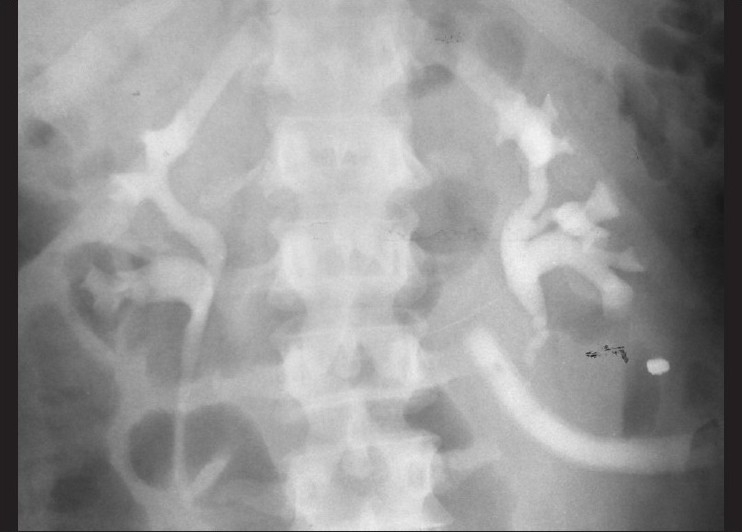
IVU, shell injury of left upper ureter, contrast pass into drain tube

**Figure 2:**
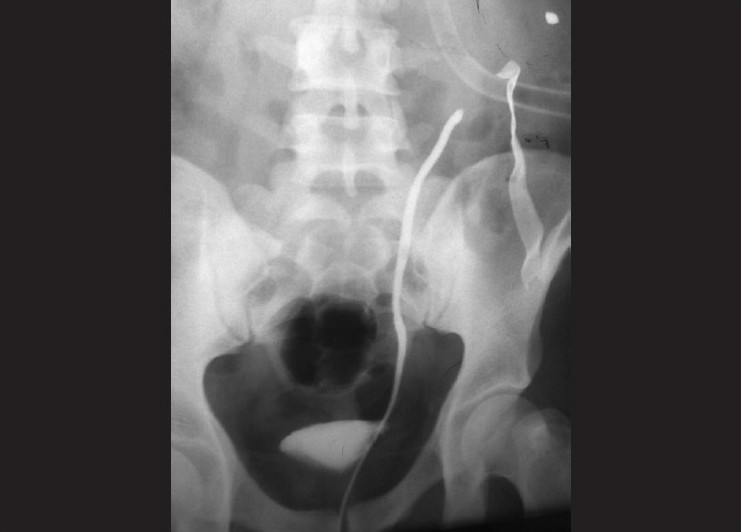
Retrograde ureterogram of the same patient revealed complete obstruction of ureter

The diagnosis of ureteric injury was made intraoperatively in eight cases (40%). While 12 cases (60%) were missed as they were discovered either in early postoperative period in the form of excess drainage fluid eight cases (66%) [Figures 1 and 2] or present lately as abdominal mass (urinoma) in four cases (34%). All patients had other associated injuries [Table 1].

**Table 1:** Associated abdominal injuries

Organ	Number of patients (%)
Small bowel	10 (50)
Colon	12 (60)
Bladder	5 (25)
Duodenum	4 (20)
Burn	4 (20)
Fracture femur	3 (15)
Spinal cord	2 (10)
Renal	2 (10)

Figures in parenthesis are in percentage

Eight ureteric injuries (40%) were of proximal one third, four (20%) were of middle one third and eight (40%) were of distal one third.

The extent or the severity of injury according to the organ injury scale was as follows. Fourteen ureteric injuries (70%) were with complete transactions, whereas six patients (30%) had injuries with with partial lesion.

**Figure 3:**
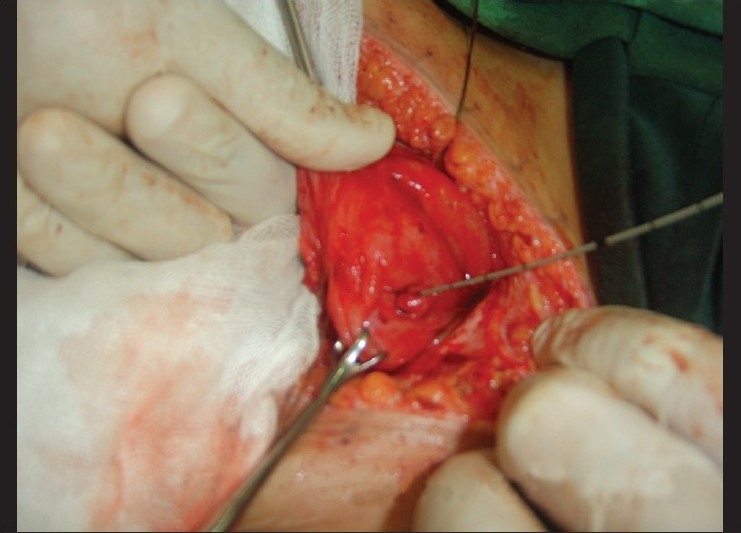
A victim of car bomb re-explored for missed lower ureteric injury treated by direct ureteroneocystostomy

Management was by end to end anastomosis in eight cases over a double J stent. Ureteroneocystostomy [Figure 3] was performed in six cases (direct anastomosis in four cases and psoas hitch anastomosis in two cases). Four cases were treated by nephrectomy (two cases were grade V renal injuries and two cases were presented with severe hydronephrosis and sepsis), and two patients were treated with ureteric stent.

Three patients (15%) died from serious associated injuries, one patient died on the fifth postoperative day and the other two patients died within 24 hours of injury.

Regarding the outcome of treated patients during the early period of follow up, eight patients had end to end anastomosis; four of them reported voiding symptoms (dysuria, urgency) that were relieved by removal of ureteric stent. One of the two patients treated with psoas hitch had developed wound dehiscence and urinary fistula, and was treated by percutaneous nephrostomy, whereas those patients who were treated by nephrectomy were doing well.

## DISCUSSION

Civil violence in Iraq has reached epidemic levels during the last 5 years, and increasing numbers of urological injuries are being seen among unprotected civilians not wearing body armor.[9]

Traumatic ureteral injuries are uncommon. The ureters are relatively well protected by the surrounding structures and their small size and mobility contributes to their infrequent injury.[2,10]

In our study, all ureteric injuries were due to either shell fragments of explosive device or gunshots 55 and 45%, respectively; no stab wounds were reported, while in other studies ureteral injuries are attributable to penetrating assault, with gunshot wounds and stab wounds accounting for approximately 81 and 16% of cases, respectively.[1,3,10]

In our study most patients underwent urgent laparotomy without further investigations. In hemodynamic stable patients, imaging studies should be conducted when urinary tract injury is suspected. Excretory urograms have low diagnostic rate for ureteric injuries reaching 40%,[1] whereas the abdominal CT scan with contrast injection and delayed scans are the gold standard for staging such injuries.[2]

Traumatic injury to the ureter is often undiagnosed at the time of presentation and may have been overlooked, due to many reasons, including the magnitude of associated injuries and low index of suspicion.[10‐14]

In our series, more than half the cases of ureteric injury were due to shell injuries from explosive devices that cause devastating damage and make the initial effort to save patient life this accounts for high number of delayed diagnosis 60%, in comparison to other series Al-ali reported that ureteral injury was missed in 47 (75%) patients of 63 war injuries.[15]

It is important to be aware of the signs of potential missed injury in the postoperative period. The most important signs of urinary leakage are prolonged ileus, low-grade fever, flank tenderness and persistent drainage from operative sites.[4,16]

In our series, all penetrating ureteric cases were associated with injuries to other organs, which coincide with the results reported widely in literature.[2,3,10,11] Associated injuries to the gastrointestinal tract are commonly present, and may modify the management of ureteral injury at initial procedure.

All patients in whom the intraoperative diagnosis was missed had colostomy and this offers two problems:

1. technical difficulty during surgery especially if ureteral injury were on the same side of colostomy
2. high risk of infection in spite of good antibiotic cover.

In our study the mortality rate was 15%, the grade of ureteric injury was not found statistically related to mortality rate, reflecting the fact that death is related to other organs injured rather than the ureter itself.

## CONCLUSIONS

The management of ureteric injuries during wars and disasters might be not ideal because the target of surgeons is to save the life of the patients within a short period of time.

In this study, a delay in diagnosis was a contributory factor in morbidity related to ureteral injury, necessitating the need for second operation in already compromised patients from associated injuries. The presence of shock on admission, delayed diagnosis and colon injuries were associated with a high complication rate.

Ureteral injuries must be considered early during the evaluation of penetrating abdominal injuries.
